# Microdamage analysis of single-use flexible ureteroscope immediately after lithotripsy use

**DOI:** 10.1038/s41598-022-23345-z

**Published:** 2022-11-01

**Authors:** Teruaki Sugino, Kazumi Taguchi, Rei Unno, Shuzo Hamamoto, Ryosuke Ando, Atsushi Okada, Takahiro Yasui

**Affiliations:** 1 Department of Nephro-Urology, Nagoya City University Graduate School of Medical Sciences, 1, Kawasumi, Mizuho-cho, Mizuho-ku, Nagoya, 467-8601 Japan; 2 Department of Urology, University of California, San Francisco, 400 Parnassus Ave, San Francisco, CA 94143 USA

**Keywords:** Urology, Medical research

## Abstract

This prospective ex vivo study investigated microdamage to single-use flexible ureteroscopes (fURS) after ureteroscopy and endoscopic combined intrarenal surgery (ECIRS). The performance of 30 WiScope devices (OTU Medical, San Jose, CA, USA) was examined immediately after use, dividing them into three equal groups: ureteroscopy and ECIRS in the prone and supine positions. The overall scope of microdamage assessment included the scope deflection, bending radius, resolution, and water flow rate. Additionally, we analyzed the association between scope status and surgical parameters. The deflection, bending radius, and resolution remained similarly above the thresholds in all groups. However, the water flow rate was below the threshold in seven scopes (70%) in the ureteroscopy group and none in the ECIRS groups (*P* = 0.001). Univariate and multivariable logistic regression analyses demonstrated that basket wire catheter use was associated with an increased risk for overall scope microdamage (odds ratio [OR], 22.70; *P* = 0.006 and OR, 22.40; *P* = 0.019, respectively). Stone size, total laser energy, and surgical position were not associated with a risk for scope microdamage. In conclusion, ureteroscopy was more closely associated with scope damage than ECIRS, and basket wire catheter use seemed to inflict more damage to the fURS.

## Introduction

The flexible ureteroscopes (fURS) technology has been developing over the recent decades^1^ As a result, fURS is widely used worldwide as first-line endoscopic management for renal or ureteral stones^2^. Its development has contributed to fewer invasive surgeries, resulting in a shorter surgery time, higher stone-free rate, and shorter hospitalization^3^.

Reusable fURS (re-fURS) is known for its significant initial purchase and maintenance costs, including cleaning and sterilization^4^. Although the reduced scope diameter during fURS development improved operability, it became more delicate, and the repair costs have increased^5^. Scope damage requiring repair occurs after about 9–12 procedures, and the scope needs frequent repairs after the first damage has occurred^6^.

Some single-use fURS (su-fURS) types have been introduced and are widely used for endoscopic management. These incur no maintenance or repair costs and provide consistent performance during surgery^4,7^. Hennessey et al. suggested that su-fURS should be used instead of re-fURS for cases that pose a high risk of scope damage, such as lower pole and staghorn stones. However, the cost-effectiveness of using su-fURS or re-URS remains debated^8–10^. To extend the life of re-fURS, we should focus on its durability and strive to suppress the costs of repair or scope replacement.

Microdamage to re-fURS that occurs during every surgery is believed to accumulate, resulting in the need for repair or poor scope performance. Understanding the microdamage caused during each surgery could help prevent major damages and reduce costs. This study evaluated the microdamage caused to su-fURS after ureteroscopy and endoscopic combined intrarenal surgery (ECIRS).

## Results

Patient characteristics are summarized in Table 1. The three groups were similar with respect to sex, age, body mass index (BMI), and stone location. The median stone size was larger, and the median average stone radiodensity was higher in the supine and prone ECIRS groups than in the ureteroscopy group (*P* < 0.001 and *P* = 0.018, respectively). The three groups had similar stone-free rates, total surgery times, ureteroscopy usage times, and total laser energy used (*P* = 0.754, 0.402, 0.717, and 0.383, respectively). A basket wire catheter was used in all patients in the ureteroscopy group and none in the ECIRS groups (*P* < 0.001).

**Table 1:** Patient characteristics.

Characteristic	Ureteroscopy	Supine ECIRS	Prone ECIRS	*P*-value
**Sex**				1
Male	6 (60%)	7 (70%)	6 (60%)	
Female	4 (40%)	3 (30%)	4 (40%)	
Age (years)^a^	62.5 (54.3–72.0)	71.5 (65.5–73.0)	70.0 (58.5–72.3)	0.535
BMI (kg/m^2^)^a^	23.8 (21.2–27.1)	23.35 (19.9–27.7)	23.9 (21.5–25.0)	0.860
**Stone location**				0.122
Kidney	4 (40%)	8 (80%)	3 (30%)	
Ureter	6 (60%)	2 (20%)	7 (70%)	
Stone size (mm^3^)^a^	1730.0 (531.0–2137.5)	5512.5 (4841.0–17,060.5)	6708.0 (3366.9–10,486.5)	< 0.001
Average stone radiodensity (HU)^a^	1120.75 (753.50–1152.50)	1323.50 (1216.17–1390.12)	1390.00 (1271.25–1522.90)	0.018
Preoperative stenting	4 (40%)	2 (20%)	2 (20%)	0.668
**Access sheath**				0.272
10/12-Fr	3 (30%)	6 (60%)	7 (70%)	
12/14-Fr	7 (70%)	4 (40%)	3 (30%)	
Stone free	8 (80%)	9 (90%)	10 (100%)	0.754
Total surgery time (min)^a^	83.5 (47.5–112.3)	102.5 (87.0–119.5)	99.5 (75.3–115.8)	0.402
Ureteroscope usage time (min)^a^	64.5 (30.5–84.8)	72.5 (61.8–86.8)	74.0 (48.3–104.5)	0.717
Total laser energy (kJ)^a^	3561.0 (231.8–24,903.3)	9247.0 (4035.0–14,058.3)	2528.0 (1502.3–4381.5)	0.383
Basket wire catheter use	10 (100%)	0 (0%)	0 (0%)	< 0.001

*ECIRS* endoscopic combined intrarenal surgery, *BMI* body mass index, *HU* Hounsfield units, *CT* computed tomography.

^a^Median (interquartile range).

Findings in the scope performance evaluation after its use are shown in Table 2 and Supplementary Tables S1–S4. Deflection failure was observed in three scopes (30%) in the ureteroscopy group and one each (10%) in the supine and prone ECIRS groups (*P* = 0.574). As shown in Supplementary Table S1, two scopes in the ureteroscopy group (20%) and one in the supine ECIRS group (10%) could not control the up and down bending of the deflection section because the deflection mechanism was severely damaged, described in the table as “not applicable.” Failure to reach the threshold bending radius was also observed in these three scopes (Supplementary Table S2). Failure to reach the resolution threshold was not observed in any of the scopes (Supplementary Table S3). As shown in Supplementary Table S4, a decrease in the water flow rate was observed in seven of the ureteroscopy group scopes (70%) and none in the two ECIRS groups (*P* = 0.001).

**Table 2:** Scope evaluation.

Variable	Ureteroscopy	Supine ECIRS	Prone ECIRS	*P*-value
Deflection failure	3 (30%)	1 (10%)	1 (10%)	0.574
Inadequate bending radius	2 (20%)	1 (10%)	0 (0%)	0.754
Insufficient resolution	0 (0%)	0 (0%)	0 (0%)	1
Decreased water flow	7 (70%)	0 (0%)	1 (10%)	0.001

Deflection and minimum bending radius were assessed in both up and down directions.

*ECIRS* endoscopic combined intrarenal surgery.

The logistic regression analysis results are shown in Table 3. Univariate and multivariate analyses revealed that basket wire catheter use was associated with an increased risk of overall scope damage (odds ratio [OR], 22.70, *P* = 0.006 and OR, 22.40, *P* = 0.019, respectively). Stone size, total laser energy, and surgical position were not associated with a risk for scope microdamage.

**Table 3:** Logistic regression analysis of factors associated with overall scope damage.

Variable	Univariate OR (95% CI)	*P*-value	Multivariable OR (95% CI)	*P*-value
Stone size	1.000 (1.000–1.000)	0.076	1.000 (1.000–1.000)	0.469
Total laser energy	1.000 (1.000–1.000)	0.075	1.000 (1.000–1.000)	0.222
Surgical position	0.111 (0.012–1.050)	0.055	0.103 (0.001–7.400)	0.297
Basket wire catheter use	22.70 (3.140–164.0)	0.006	22.40 (1.660–300.0)	0.019

*OR* odds ratio, *CI* confidence interval.

## Discussion

The financial burden on urolithiasis management is substantially increased by the costs of re-fURS maintenance and repair^11^. This study investigated the microdamage caused to su-fURS during ureteroscopy and ECIRS surgeries. Our results showed that ureteroscopy was more closely associated with scope damage than ECIRS, as was basket wire catheter use. In contrast, stone size, total laser energy, and surgical position were poorly associated with scope microdamage. These findings could help optimize the urolithiasis treatment by selecting the appropriate fURS for each procedure. Moreover, they suggest that we should be careful with possible scope damage when removing fragments using a basket wire catheter.

Our study demonstrated that ureteroscopy tended to inflict slightly more microdamage to the scope deflection mechanism than did ECIRS. Hosny et al. reported that the fURS deflection tip was one of the scope’s most fragile parts^12^. Excessive stress on the deflection mechanism decreases the deflection angle^13^. Applying excessive force to bend the scope tip in the pelvis or careless processing of the scope through the access sheath could damage the deflection mechanism^14,15^. We initially hypothesized that the fURS was more likely to be damaged during ECIRS than during ureteroscopy because of the larger stones in the former. However, ureteroscopy seems to cause more damage to the scope than ECIRS. This might be because frequent insertions of the laser fiber, basket wire catheter, and fURS into the access sheath were necessary to collect stone fragments during ureteroscopy. In contrast, stone fragments were collected by retrograde irrigation through the fURS in ECIRS. Proper access sheath use during ureteroscopy is essential to reduce scope damage^16^. A large access sheath diameter may reduce damage to the ureteroscope, especially to the deflection mechanism. However, in the current study, a 12/14-Fr access sheath was used in two patients with deflection failure in the ECIRS group, while a 10/12-Fr UAS was used in three patients with deflection failure in the ureteroscopy group, indicating that in the prevention of damage to the ureteroscope, access sheath usage is more important than access sheath diameter. Some scopes, including the WiScope (OTU Medical, San Jose, CA, USA) used in this study, cannot be automatically straightened when the articulation lever is released. The highly damaged up-and-down deflection mechanism observed in two of the scopes used for ureteroscopy in this study occurred because the scopes were removed through the access sheath without straightening. These scope types must be consciously straightened during insertion or removal.

The WiScope (OTU Medical), a digital fURS, has a better image quality than fiber optic fURS but no additional benefit in terms of scope durability and surgical performance^17,18^. The resolution failure after their use has not been investigated before, although studies comparing the resolution between scopes before surgical use are available (e.g., su-fURS vs. re-fURS)^19^. Our study demonstrated that single surgical use did not cause significant damage to scope resolution, regardless of the operation type.

We alternately inserted the catheter and the laser fiber during stone fragment collection with a basket wire catheter. Seto et al. reported that repeated insertions of these accessories cause damage to the fURS working channel, resulting in decreased water flow rate^20^. We believe that the size of the basket wire catheter may influence scope damage; a larger catheter may decrease the water flow rate to a higher extent. However, the water flow rate decreased in 70% of the patients for whom the basket catheter was used, even though we used the thinnest 1.5-Fr basket wire catheter. Thus, we believe that, in addition to the thickness of the catheter, the number of basket wire catheter insertions is an important factor that causes damage to the working channel. In patients with a decreased water flow rate, the average number of basket wire catheter insertions was 3.4. In three patients without a decreased water flow rate for whom a basket wire catheter was used, it was inserted only once because the stone size was small. Seto et al. also indicated that scope deflection with a basket wire catheter in the channel does not cause significant damage to the scope despite using a deflection angle of over 120°. However, deflection of scopes with 200 μm holmium laser fibers in the channel could cause visible damage to the channel when the deflection angle is over 60°. Therefore, the scope must be straightened when inserting or removing the laser fibers. Moreover, su-fURS might be better than re-fURS for ureteroscopy because alternate insertion of a basket wire catheter and the laser fiber is needed, particularly in cases with large or impacted stones that could damage the scope.

We found no association between the total laser energy and microdamage to fURS. Thermal laser damage to fURS is common, frequently occurring approximately 3–4 mm from the scope tip^5^. It is essential to advance the laser fiber tip to one-quarter of the screen (3 mm or more from the scope tip) during fragmentation to avoid thermal damage^21^. This safe distance could reduce the damage caused by the plasma bubbles generated by the laser fiber tip, even when high-energy settings are used. We always attempt to maintain a safe distance during stone fragmentation, which may explain the weak association between the damage and total laser energy.

The limitations of the current study include the relatively small number of scopes assessed, which might have resulted in an underpowered study. Additionally, we investigated the fURS microdamage using only one scope type; therefore, the results might not apply to other fURS types, such as re-fURS and su-fURS other than WiScope (OTU Medical). Moreover, surgeries were performed by different surgeons, which might have affected the results. Additionally, the status of the scopes was not evaluated immediately before surgery. The scopes were checked to see if they satisfied the pass criteria before they were shipped; however, it is possible that defective products were shipped erroneously. Despite these limitations, the use of su-fURS in this study allowed a unique evaluation of the scope status immediately after its surgical use. Our data could contribute to the reduction of damage caused to re-fURS, resulting in the extension of its life and reducing the costs. Furthermore, our study supports the choice of fURS (su-fURS or re-fURS) in ureteroscopy and ECIRS.

## Conclusions

We investigated the microdamage caused during surgery to su-fURS. Ureteroscopy was more closely associated with scope damage than ECIRS. Basket wire catheter use was associated with scope damage, while the stone size, total laser energy, and patient position were not. These results help better understand the microdamage caused during each surgery, which could help prevent major damages and reduce costs.

## Patients and methods

### Patients

We recorded the patient sex, age, BMI, stone location, stone size (mm^3^), and average stone radiodensity preoperatively. Moreover, we evaluated surgical parameters, including total surgical time, ureteroscope usage time, total laser energy, stone-free rate, and use of a basket-wire catheter. Stone-free status was defined as no residual stones or stones smaller than 4 mm in diameter, as determined by plain abdominal radiography 3 months postoperatively. Patients with a single kidney, urinary diversion, age < 20 years, or medical history of ureteroscopy or ECIRS were excluded from this study.

### Study design

The Institutional Review Board of Nagoya City University Hospital approved this ex vivo study before it started (60-19-0044). The study followed the tenets of the Declaration of Helsinki. All patients provided informed consent to participate in the study.

**Figure 1:**
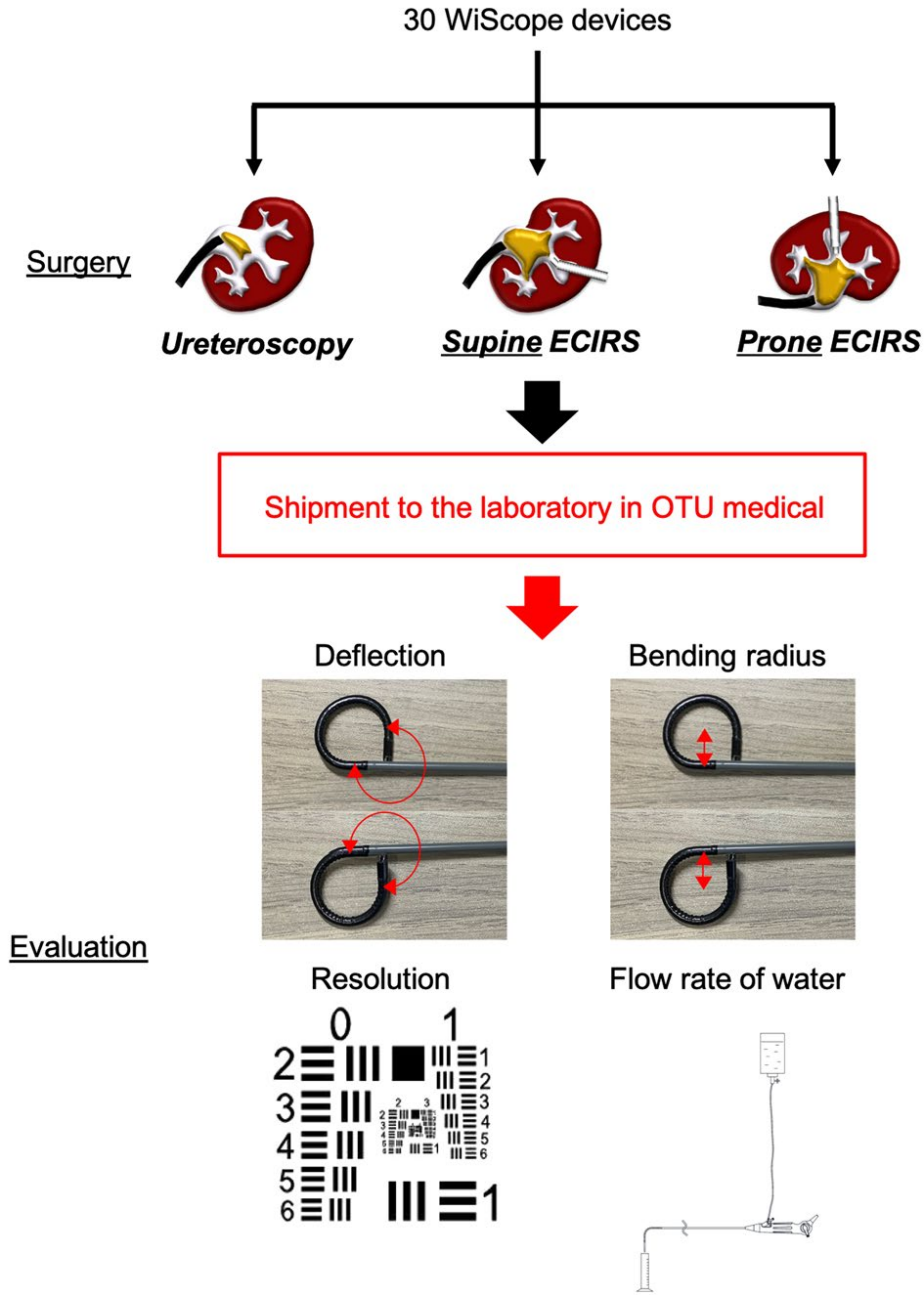
Study design. This prospective ex vivo study analyzed the performance of 30 WiScope devices (OTU Medical, San Jose, CA, USA) immediately after their use. We included scopes used for ureteroscopy and ECIRS in the prone and supine positions, ten each. All scopes were sent after a single surgical use to OTU Medical for testing and damage assessment.

The study design is summarized in Fig. 1. We assessed the performance of 30 WiScope devices (OTU Medical) immediately after use. The scopes were divided into three equal groups: ureteroscopy and ECIRS in the prone and supine positions. Ureteroscopy was performed in patients with proximal ureteral stones < 10 mm and kidney stones < 20 mm in diameter; ECIRS was performed for patients with larger proximal ureteral stones (> 10 mm) and kidney stones (> 20 mm). Patients with 10–20-mm lower pole stones were excluded from this study. The surgical position was randomly determined in ECIRS. The scopes were sent to the laboratory at OTU Medical after use to evaluate their deflection, bending radius, resolution, and water flow rate.

### Surgical techniques

All patients were treated under general anesthesia. A 0.035-inch guidewire was inserted through the ureteral orifice followed by a 10/12-Fr or 12/14-Fr access sheath. In the ureteroscopy group, retrograde fragmentation was performed using a 272-μm holmium YAG laser (Cyber Ho, Quanta System, Milan, Italy), and the fragments were removed using a 1.5-Fr basket wire catheter (NCircle, Cook Medical, Bloomington, IN, USA). In the ECIRS groups, percutaneous access was established using a 16/17.5-Fr miniature percutaneous nephrolithotomy tract (Karl Storz, Tuttlingen, Germany). Two urologists simultaneously fragmented the stones, one by antegrade fragmentation using LithoClast lithotripsy (Electro Medical Systems S.A., Nyon, Switzerland) with a 12-Fr mini-nephroscope (Karl Storz), and the other by retrograde fragmentation using a holmium YAG laser with fURS. The fragments were washed through the nephrostomy sheath using retrograde irrigation.

### Postoperative scope microdamage evaluation

The scope deflection, bending radius, resolution, and water flow rate were assessed to determine whether the postoperative status exceeded the pass criteria before shipping, as shown in Supplementary Tables S1–S4. The status of each scope was evaluated as follows:

#### Deflection

1. The deflection section was bent to its utmost up and down positions by pushing the articulation level of the scope without any accessories in the working channel.
2. The angle was measured using a digital protractor.

#### Bending radius

1. The deflection section was bent to its utmost up and down positions by pushing the articulation level of the scope without any accessories in the working channel.
2. The radius was measured using a digital caliper.

#### Water flow rate

1. One end of the tube was inserted into a 500-mL normal saline bottle, and the other end was connected to an irrigation port of the scope. The accessory port was sealed with a cap.
2. The 500-mL normal saline bottle was hanged vertically 100 cm above the scope.
3. The scope was held in a horizontal position, and the valve opened.
4. At least 15 s were allowed to ensure uninterrupted saline solution flow.
5. The amount of saline flowing in one minute was measured.

#### Resolution

1. A 1951 U.S. Air Force (USAF) resolution test chart (Fig. 1) was placed underneath the distal tip and parallel to it.
2. The distance between the tip and target was adjusted to 10 mm with a vernier caliper, and distortion was checked using distortion grid target cards.
3. The resolution was recorded in line pairs per millimeter (LP/mm) and determined using a reference chart included in the test target.

### Statistical analysis

Data are presented as numbers (%) or medians (interquartile ranges) and analyzed using EZR for R (R project 3.6.3)^22^. Fisher’s exact test and the Mann–Whitney *U* test were used to compare the ureteroscopy and ECIRS groups. The Kruskal–Wallis test was used to compare the three groups. Moreover, logistic regression analysis was performed to investigate the association between the overall scope damage (deflection, bending radius, resolution, and water flow rate) and other variables such as stone size, total laser energy, surgical position, and the use of a basket wire catheter. Statistical significance was set at *P* < 0.05.

### Ethical approval

The present study was approved by the Institutional Review Board of Nagoya City University Hospital (60-19-0044). The study followed the tenets of the Declaration of Helsinki. All participating patients provided informed consent for the use of their data.

## Supplementary Information

Supplementary Tables.

## Data Availability

All data generated during this study are included in this published article. They are available from the corresponding author on reasonable request.
