# Supplementary material for: Microdamage analysis of single-use flexible ureteroscope immediately after lithotripsy use
Source: Sci Rep. 2022 Nov 1;12:18367. doi: 10.1038/s41598-022-23345-z (PMC9626578; doi:10.1038/s41598-022-23345-z)
Supplement: Supplementary file 1 — Supplementary Tables. [file 41598_2022_23345_MOESM1_ESM.pdf]

Microdamage analysis of single-use flexible ureteroscope immediately after lithotripsy use

Teruaki Sugino, Kazumi Taguchi, Rei Unno, Shuzo Hamamoto, Ryosuke Ando, Atsushi Okada, Takahiro Yasui

Supplementary Table S1. Detailed data on the scope deflection evaluation

Operation type	Ureteroscopy		Supine ECIRS		Prone ECIRS	
Direction	Up	Down	Up	Down	Up	Down
Pass criteria	≥ 275°	≥ 275°	≥ 275°	≥ 275°	≥ 275°	≥ 275°
Scopes	not applicable	282.8	298.9	299.8	300.7	299.4
	309.0	290.6	304.7	301.5	293.3	290.4
	287.5	293.5	301.8	305.6	263.0	284.1
	292.3	284.9	295.5	298.5	301.1	304.8
	202.8	261.6	not applicable	not applicable	300.3	302.7
	296.1	315.3	301.1	302.3	305.7	308.5
	not applicable	284.8	302.2	296.9	298.6	281.8
	308.7	295.2	310.1	299.5	305.4	292.4
	300.9	295.0	297.5	297.3	299.4	303.2
	306.3	303.1	302.2	298.9	294.8	305.4
Failure	3 (30%)		1 (10%)		1 (10%)	
P-value	0.574					

ECIRS, endoscopic combined intrarenal surgery.

Supplementary Table S2. Detailed data on the scope bending radius evaluation

Operation type	Uteteroscopy		Supine ECIRS		Prone ECIRS	
Direction	Up	Down	Up	Down	Up	Down
Pass criterion	8 ± 0.5 mm	8 ± 0.5 mm	8 ± 0.5 mm	8 ± 0.5 mm	8 ± 0.5 mm	8 ± 0.5 mm
Scopes	not applicable	8.27	7.76	7.81	7.86	7.89
	7.96	8.44	7.73	8.06	7.80	7.94
	8.11	8.16	7.79	7.78	8.21	7.84
	8.41	8.32	7.71	7.82	7.68	7.68
	7.65	8.15	not applicable	not applicable	8.05	8.04
	7.75	7.99	7.69	7.87	7.62	7.79
	not applicable	7.96	8.02	8.11	7.59	7.68
	7.69	7.84	7.93	8.19	7.84	7.81
	7.86	7.84	7.83	7.92	7.78	7.74
	7.58	7.79	7.89	8.13	7.78	7.91
Failure	2 (20%)		1 (10%)		0 (0%)	
P-value	0.754					

ECIRS, endoscopic combined intrarenal surgery.

Supplementary Table S3. Detailed data on the scope resolution evaluation

Operation type	Ureteroscopy	Supine ECIRS	Prone ECIRS
Direction			
Pass criterion	≥ 4 LP/mm	≥ 4 LP/mm	≥ 4 LP/mm
Scopes	5.04	4.49	4.49
	4.49	5.04	5.04
	5.04	4.49	5.04
	4.49	5.04	5.04
	4.49	4.49	4.49
	4.49	5.04	5.04
	4.49	5.04	4.49
	4.49	5.04	4.49
	5.04	5.04	5.04
	5.04	5.04	5.04
Failure	0 (0%)	0 (0%)	0 (0%)
P value	1		

ECIRS, endoscopic combined intrarenal surgery; LP, line pairs.

Supplementary Table S4. Detailed data on the scope water flow rate evaluation

Operation type	Ureteroscopy	Supine ECIRS	Prone ECIRS
Direction			
Pass criterion	≥ 35 mL/min	≥ 35 mL/min	≥ 35 mL/min
Scopes	34	36	42
	34	42	34
	32	38	38
	34	42	40
	26	42	42
	32	44	42
	34	42	42
	44	44	42
	34	44	44
	40	46	42
Failure	7 (70%)	0 (0%)	1 (10%)
P-value	0.001		

ECIRS, endoscopic combined intrarenal surgery.
